# Clinical Factors Influencing Tacrolimus Metabolism and Blood Level Early After Kidney Transplantation—A Comparison of Three Different Tacrolimus Formulations

**DOI:** 10.3390/jcm14124223

**Published:** 2025-06-13

**Authors:** Aureliusz Kolonko, Andrzej Więcek

**Affiliations:** Department of Nephrology, Transplantation and Internal Medicine, Medical University of Silesia, 40-027 Katowice, Poland; awiecek@sum.edu.pl

**Keywords:** body composition, early conversion, intestinal permeability, tacrolimus formulation

## Abstract

**Background:** Optimal tacrolimus dosing in the early post-transplant period is still challenging. We prospectively studied the possible associations between selected parameters of recipient body composition, markers of intestinal permeability and tacrolimus dosing and blood level in kidney transplant recipients (KTRs) treated with three different tacrolimus formulations. **Methods:** When discharged from hospital immediately after kidney transplantation, markers of intestinal permeability, body composition parameters and tacrolimus blood level were assessed in 165 KTRs treated with Prograf, Advagraf or Envarsus. **Results:** In the stepwise multivariate analysis performed in patients treated with Prograf, only age independently influenced the tacrolimus exposure expressed as area under the curve (AUC). In patients treated with Advagraf, eGFR (r_partial_ = 0.291; *p* < 0.05), antithymocyte globulin (vs. basiliximab) induction (r_partial_ = 0.445; *p* < 0.001), lipopolysaccharide (LPS) level (r_partial_ = 0.393; *p* < 0.01) and drug dose (r_partial_ = 0.433; *p* < 0.01) were independently associated with tacrolimus AUC. In patients treated with Envarsus, only age (r_partial_ = −0.365; *p* < 0.05) and fatty-acid-binding protein (FABP-2) level (r_partial_ = −0.364; *p* < 0.05) were independently associated with the tacrolimus AUC. **Conclusions:** We confirmed the significant association between markers of intestinal permeability and tacrolimus exposure in KTRs who underwent early post-transplant conversion from Prograf to Advagraf or Envarsus. This may suggest that the planned tacrolimus conversion from the twice-daily to the once-daily formulation should be performed later (at least 3 months after transplantation) to avoid unnecessary tacrolimus blood level instability.

## 1. Introduction

Today, in a majority of kidney transplant recipients (KTRs), the immunosuppressive regimen is based on tacrolimus. As it is characterized by considerable blood level variability and a narrow therapeutic index, its appropriate dosing is still challenging, especially in the early period after transplantation [1,2]. Moreover, during the last few decades, the innovative once-daily tacrolimus formulations characterized by different mechanisms of drug extended release were successfully introduced. Importantly, these novel tacrolimus formulations present different pharmacokinetics, which makes the precise dosing even more difficult [2,3,4].

Until now, several clinical factors had been identified as modifying the tacrolimus blood level shortly after transplantation, including recipient age, body mass index (BMI), CYP3A4 polymorphism [5,6], the presence of anti-HCV antibodies [6], hematocrit [7] and comedication [8]. Additionally, the potential relationship between several parameters of body composition and tacrolimus pharmacokinetics was previously investigated [9,10]. In our recent analysis, the pre-transplant lean body mass index (LBMI) assessed by bioimpedance was shown to be a potentially useful tool for the optimization of initial tacrolimus dosing [11].

Finally, it is worth noting that diverse twice-daily and once-daily tacrolimus formulations ex definitione differ substantially in regard to the intestinal absorption site and efficacy [12]. Various disturbances of gastrointestinal tract function, frequently observed during the early post-operative days, significantly modify the intestinal permeability and tacrolimus bioavailability [13]. On the other hand, those disturbances may be further augmented by the immunosuppressive drugs [14]. Furthermore, during the last decade, it was shown that gut microbiota structure could be altered after kidney transplantation and linked to some post-transplant complications, like diarrhea or acute rejection [15]. Importantly, some microbiota species were associated with tacrolimus dosing and exposure in the first post-transplant month [16]. To date, several biomarkers were proposed to assess the intestinal permeability and gut barrier injury, including fatty-acid-binding protein 2 (FABP-2), lipopolysaccharide (LPS), LPS-binding protein (LBP) and zonulin [17,18,19,20], although their usefulness is still uncertain. However, both FABP-2 and LPS levels seemed to be reliable measures of intestinal permeability in the KTRs cohort [21].

Based on all the abovementioned evidence, we prospectively investigated the potential associations between recipient body composition parameters, intestinal permeability markers and early post-transplant tacrolimus dosing and exposure in patients treated with three different tacrolimus formulations.

## 2. Materials and Methods

### 2.1. Study Group

The analyzed cohort included 165 unselected patients, who were prospectively recruited and studied at the time of cadaveric kidney transplantation in our center. During the first post-transplant hospital stay, patients were divided into three study groups: group 1 (discharged on Prograf^®^, Astellas Pharma Inc., Chuo City, Japan, n = 56), group 2 (converted to and discharged on Advagraf^®^, Astellas, n = 59) and group 3 (converted to and discharged on Envarsus^®^, Chiesi, Parma, Italy, n = 50). In each patient, the tacrolimus exposure was assessed at hospital discharge, using blood trough and 3h post-dose levels, the calculated area under the curve (AUC) and the tacrolimus concentration-to-dose (CD) ratio. On the same day, bioimpedance analysis of body composition was performed. Blood samples were also collected to determine markers of intestinal permeability.

The study was conducted in concordance with the protocol of Helsinki. The Institutional Review Board accepted the study protocol (No KNW/0022/KB1/122/19, BNW/NWN/0052/KB/27/24, accept date: 12 November 2019), and all participants gave written informed consent.

### 2.2. Immunosuppression Protocol

Before transplantation, all patients received a triple immunosuppressive regimen, which consisted of twice-daily tacrolimus (Prograf), mycophenolate mofetil and steroids. Induction therapy was also prescribed in all subjects, using basiliximab (Simulect^®^, Novartis, Nurnberg, Germany, n = 105) or antithymocyte globulin (Thymoglobulin^®^, Genzyme Europe, Naarden, Holland, n = 61). After the second tacrolimus through level measurement, at a median of 8 (7–9) days post-transplant, patients from study groups 2 and 3 were converted in a randomized manner to Advagraf (with the conversion rate 1:1) or Envarsus (with the conversion rate 1:0.7), respectively, whereas patients in group 1 remained on Prograf therapy.

### 2.3. Bioimpedance Analysis

Body composition analysis was performed on the day of discharge from the hospital using a BIA device (InBody 770, InBody Japan Inc., Tokyo, Japan) with a multifrequency analyzer (1, 5, 50, 500 and 1000 kHz), according to the manufacturer’s instructions. Intracellular water (ICW), extracellular water (ECW), total body water (TBW), ECW/TBW ratio, phase angle, visceral fat area (VFA, expressed in cm^2^), lean body mass (LBM) and lean body mass index (LBMI, expressed in kg/m^2^) were calculated.

### 2.4. Laboratory Parameters

Plasma levels of IL-6 and bacterial lipopolysaccharides (Uscn Life Sciences Inc., Wuhan, China) were assessed by ELISA, with the intra-assay and inter-assay coefficients of variability <7.2 and <7.8%, and <10 and <12%, respectively. Plasma FABP-2 (R&D systems Inc., Minneapolis, MN, USA) and LBP levels (CloudClone Corp, Katy, TX, USA) were measured by an ELISA kit with the intra-assay and inter-assay coefficients of variability <4.1 and 11.1%, and <10 and <12%, respectively.

### 2.5. Statistical Analysis

Statistical analyses were performed using Statistica 13.3 PL for Windows (Tibco Inc., Palo Alto, CA, USA) and MedCalc v20.014 (MedCalc Software, Mariakerke, Belgium). Values are presented as means with a 95% confidence interval, medians with interquartile ranges or frequencies. The main study comparison was performed between 3 groups of patients based on different tacrolimus formulations, using the ANOVA (for quantitative variables) or the χ2 test (for qualitative variables). Variables with a non-normal distribution were compared using the Kruskal–Wallis test. For further between-group comparisons, the Mann–Whitney U-test was used. Calculation of correlations was performed using the Spearman coefficient. Finally, the stepwise multiple regression analyses were performed separately for all three tacrolimus formulations (Prograf, Advagraf and Envarsus) with the tacrolimus AUC at hospital discharge as a dependent variable. The potential independent variables for each multivariate analysis were selected based on the results of univariate analyses. For all analyses, a *p* value below 0.05 was considered statistically significant.

## 3. Results

### 3.1. Study Group Characteristics

The study cohort consisted of 165 kidney transplant recipients, divided into three groups, based on the different tacrolimus formulations (Prograf, Advagraf or Envarsus). The clinical characteristics of study groups are presented in Table 1. There were no significant differences in basic demographic and clinical parameters, including pre-transplant residual diuresis and dialysis vintage. Patients from the Prograf group had a significantly lower BMI and more often received antithymocyte globulin vs. basiliximab induction in comparison with the Advagraf or Envarsus groups. The primary kidney disease diagnosis did not differ between groups (*p* = 0.54). The length of hospital stay [14 (13–18 days)] was also comparable between groups (*p* = 0.23). There were no significant differences in systolic and diastolic blood pressure values and serum creatinine concentration between study groups on the day of the discharge from the hospital.

### 3.2. Pre-Transplant Factors and Tacrolimus Exposure at Hospital Discharge

As expected, the tacrolimus dosing was significantly lower and the C/D ratio greater in the Envarsus group as compared to the other study groups (Table 1). Importantly, despite the lower 3h post-dose blood level in this group, the discharge tacrolimus AUC was comparable in all three study groups.

In the whole study cohort, the daily dose of tacrolimus (R = −0.332), dose per kg of body weight (R = −0.392) and AUC (R = −0.262) were significantly associated with recipient age (all *p* < 0.001) (Table 2). When study groups were analyzed separately, recipient age was significantly associated with tacrolimus daily dose in both once-daily formulation groups (Advagraf, R = −0.344; *p* < 0.01; Envarsus, R = −0.599; *p* < 0.001) but not in the Prograf group (*p* = 0.26). Moreover, the universal association of recipient age with tacrolimus dose/kg of body weight seems to be much stronger in the Envarsus group (R = −0.603; *p* < 0.001) than in the two other groups (Prograf, R = −0.297; *p* < 0.05; Advagraf, R = −0.397; *p* < 0.01). On the other hand, the AUC was influenced by recipient age in the Prograf group (R = −0.302; *p* < 0.05) and the Advagraf group (R = −0.269; *p* < 0.05) but not in the Envarsus group (Table 2).

It is of interest that while the amount of pre-transplant residual diuresis or cold ischemia time did not affect the tacrolimus exposure, the time of pre-transplant dialysis therapy was negatively correlated with tacrolimus 3h post-dose level (R = −0.406; *p* < 0.01) and AUC (R = −0.316; *p* < 0.05), but only in the Envarsus group. Similarly, both tacrolimus daily and per-kg dosing (R = −0.460 and R = −0.483, retrospectively; both *p* < 0.001) as well as C/D ratio (R = −0.327; *p* < 0.05) in the latter group were negatively influenced by donor age.

### 3.3. The Body Composition Parameters and Tacrolimus Dosing and Exposure

No significant differences were found between the study groups in terms of body composition parameters (Table 3).

As shown in Table 4, the amount of both ECW and ICW positively correlated with tacrolimus daily dose and negatively with the C/D ratio only in the Prograf group, while they correlated negatively with dose per kg in the Advagraf group. Similar relationships were observed for the LBMI. Notably, no such associations were noted in the Envarsus group.

In contrast, the percentage of LBM was positively associated with tacrolimus dose per kg in all analyzed groups, while VFA was negatively associated, and these associations were stronger for VFA (Table 4).

Importantly, except the negative influence of the ECW/TBW ratio on the Prograf 3h post-dose level and AUC and the weak positive association of the phase angle on the Prograf 3h post-dose level, any other analyzed body composition parameter did not affect tacrolimus exposure measures (Table 4).

### 3.4. Markers of Intestinal Permeability and Tacrolimus Exposure

At hospital discharge, median FABP-2 and LBP blood levels were significantly higher in the Prograf group as compared to the Envarsus group (Table 3), whereas median LPS concentrations were comparable in all three study groups.

In the Prograf group, no significant correlation was noted between any analyzed marker of intestinal permeability and neither tacrolimus dosing nor tacrolimus exposure measures. In the Advagraf group, there were weak positive associations between LPS concentration and both tacrolimus 3h level (R = 0.270; *p* < 0.05) and AUC (R = 0.299; *p* < 0.05). In contrast, in the Envarsus group, FABP-2 concentration was significantly negatively associated with tacrolimus exposure (trough: R = −0.296; *p* < 0.05, 3h: R = −0.394; *p* < 0.01, AUC: R = −0.375; *p* < 0.01). Additionally, LPS concentration was significantly positively associated with tacrolimus AUC (R = 0.319; *p* < 0.05).

### 3.5. The Results of Multivariate Analyses

In the Prograf group, the stepwise multivariate analysis model including recipient age, ECW/TBW ratio and tacrolimus dose showed that only age independently negatively influenced the tacrolimus AUC (r_partial_ = −0.333; *p* < 0.05) (with a coefficient of determination R2 value of 0.11).

In the Advagraf group, the initial model included recipient age, eGFR, LPS level, LBP level, tacrolimus dose and the type of induction therapy. The multivariate analysis revealed that eGFR (r_partial_ = 0.291; *p* < 0.05), antithymocyte globulin (vs. basiliximab) induction (r_partial_ = 0.445; *p* < 0.001), LPS level (r_partial_ = 0.393; *p* < 0.01) and tacrolimus dose (r_partial_ = 0.433; *p* < 0.01) were independently positively associated with tacrolimus AUC (R2 = 0.39). Notably, an analogic analysis in the homogenous subgroup of patients treated only with basiliximab induction (N = 40) yielded similar results [eGFR (r_partial_ = 0.526; *p* < 0.001), LPS level (r_partial_ = 0.477; *p* < 0.01) and tacrolimus dose (r_partial_ = 0.335; *p* < 0.05)], with the R2 value of 0.45.

In the Envarsus group, the model included recipient age, BMI, tacrolimus dose and FABP-2 level. The multivariate analysis revealed that only age (r_partial_ = −0.365; *p* < 0.05) and FABP-2 level (r_partial_ = −0.364; *p* < 0.05) were independently negatively associated with the tacrolimus AUC (R2 = 0.22).

### 3.6. The Tacrolimus Trough Level Variability After Conversion to Once-Daily Formulation

In both conversion groups (Prograf to Advagraf and Prograf to Envarsus group) the drug conversion was performed at a similar post-transplant time [median 8 (7–9) days vs. 8 (7–10) days, respectively; *p* = 0.48]. Prior to conversion, the tacrolimus trough levels (maintained using Prograf formulation) were comparable in both groups [8.9 (6.8–11.2) vs. 8.2 (6.4–11.4) ng/mL, respectively; *p* = 0.64]. Notably, after the conversion, a significantly higher tacrolimus trough level was noted in the Envarsus group 2 days [median 9.9 (7.7–12.5) vs. 8.6 (6.7–10.2) ng/mL; *p* < 0.05] and 4 days [median 9.8 (8.2–13.5) vs. 8.3 (7.1–9.7) ng/mL; *p* < 0.01] after conversion. The percentage of post-conversion tacrolimus trough levels > 11 ng/mL was significantly greater in the Envarsus group than in the Advagraf group, both at day 2 (34 vs. 17%; *p* < 0.05) and day 4 (43.2 vs. 11.4%; *p* = 0.001) post-conversion. Even at hospital discharge, this difference was of borderline significance [median 9.3 (7.9–11.6) vs. 8.4 (7.2–9.7) ng/mL; *p* = 0.063].

## 4. Discussion

In the present study, we found substantial differences in clinical factors associated with tacrolimus exposure prior to hospital discharge between the groups of patients treated with three different tacrolimus formulations. The analysis comprises demographic and transplant procedure data as well as various recipient body composition parameters and intestinal permeability marker levels. We found that for the twice-daily tacrolimus formulation (Prograf), only the recipient’s age influenced the AUC, whereas in the case of both analyzed once-daily formulations, the AUC was also associated with markers of intestinal permeability.

The rationale for such an investigation comes from different pharmacokinetics of all three analyzed drug formulations. Prograf is absorbed mainly in the upper intestine, whereas the tacrolimus granulate formulation (Advagraf) demonstrates prolonged drug release (90% absorption after 6–12 h) [2]. The third formulation, Envarsus, uses the unique MeltDose technique and is characterized by the progressive drug release in the distal intestine [22]. It increases drug bioavailability and decreases drug costs [23] but could also escalate the potential effect of frequently seen disturbances of the gastrointestinal tract function on tacrolimus blood level, which might lead to its excessive variability while using any of the once-daily formulations [24]. Notably, this makes it difficult to promptly achieve and further maintain the target tacrolimus blood concentration and may require more sequential blood level measurements to accomplish this goal. It would be of special importance during the very first post-transplant weeks, as the antibiotics administration was shown to increase the tacrolimus C/D ratio and tacrolimus trough level variability [25]. Moreover, it should be remembered that the uremic milieu itself augments the intestinal permeability in the experimental models [26]; thus, patients with delayed graft function are more prone to the risk of tacrolimus overexposure.

Concisely, in Advagraf-treated patients, the tacrolimus exposure expressed as AUC in both multivariate models was positively modified by eGFR, LPS level and tacrolimus dose, whereas in KTRs receiving Envarsus, only age and FABP-2 negatively influenced the tacrolimus AUC. Taking into account that the LPS level may reflect not only the loss of intestinal epithelium integrity but also the intensity of post-operative inflammation [27], these results do suggest a closer relationship between the intestinal permeability and tacrolimus exposure in the Envarsus-treated patients. Notably, the greater tacrolimus AUC prior to hospital discharge in the patients using antithymocyte globulin versus basiliximab induction probably results from the recent cessation of routine fluconazole prophylaxis in the ATG-treated group.

In our present study, we noticed significant differences in tacrolimus blood levels during the first week post-conversion from twice-daily to once-daily formulation, with inappropriately higher blood levels in the Envarsus group as compared with the Advagraf group, leading to the prolonged time to the target tacrolimus post-conversion level achievement in the former group. This may be a consequence of greater dependence of tacrolimus levels upon the gastrointestinal tract wall injury in Envarsus-treated subjects and may lead to the further exaggeration of bowel disturbances, as the preferential absorption sites were experimentally shown in the proximal and distal tract for Advagraf and Envarsus, respectively [28]. This may also suggest performing early Prograf-to-Envarsus conversion using a lower conversion rate than the one established in the stable patients [29]. However, taking into account that such an early conversion during the first post-transplant weeks requires an adjustment for the improving kidney graft function, fluctuating hemoglobin level, comedication and, of course, non-optimal pre-conversion tacrolimus level, it makes this procedure very complicated, and it would be better to plan it for the following months.

It is worth noting that in the present study, we concurrently investigated several body composition parameters as potential factors influencing tacrolimus exposure, but such an association was not confirmed in the multivariate analysis models. Interestingly, the lean body mass index was previously shown to influence the tacrolimus exposure during the first post-transplant days [11]. Such a discrepancy may suggest that within the first few post-transplant weeks, the frequently observed dysfunction of the gastrointestinal tract as well as the intestinal absorption abnormalities gain the predominant impact on the subsequent tacrolimus exposure, which covers the importance of other possibly relevant factors.

The limitations of the current study include the limited number of participants and the lack of CYP3A5 genotyping. We also did not collect the fecal specimens to determine other markers of intestinal permeability or microbiota.

## 5. Conclusions

In the present study, we confirmed the significant influence of markers of intestinal permeability on the tacrolimus exposure in KTRs who underwent early post-transplant conversion from Prograf to Advagraf or Envarsus. Taking into account that a substantial percentage of KTRs are supposed to have different disturbances of gastrointestinal tract function during the first post-operative weeks, we believe that the planned conversion of tacrolimus from the twice-daily to the once-daily formulation should be performed later, perhaps from the third month of follow-up, to avoid unnecessary overexposure to tacrolimus and instability of tacrolimus blood levels.

## Figures and Tables

**Table 1:** Clinical characteristics of kidney transplant recipients treated with different tacrolimus formulations.

	Prograf N = 56	Advagraf N = 59	Envarsus N = 50	*p*
**Recipient**				
Age [years]	50 (47–53)	50 (47–54)	49 (46–52)	0.88
Sex (M/F)	35/21	35/24	29/21	0.89
BMI * [kg/m^2^]	24.2 (21.9–26.6)	25.2 (23.0–29.6)	25.9 (23.3–29.0)	<0.05
Dialysis vintage * [months]	34 (21–55)	33 (21–45)	31 (22–45)	0.71
Residual diuresis * [mL]	300 (25–1000)	500 (100–1500)	500 (100–1200)	0.85
**Transplant procedure**				
Donor age * [years]	46.5 (36.5–57.0)	50.0 (39.0–59.0)	50.0 (37.0–57.0)	0.39
Retransplant [%]	19.6	13.6	6.0	0.12
Induction therapy [%]: IL-2 RB ATG				
	48.2	72.9	66.0	<0.05
	51.8	27.1	34.0	
CIT [h]	18 (16–19)	17 (15–19)	19 (17–21)	0.22
DGF [%]	16.1	23.7	20.0	0.59
Length of hospital stay [days]	13 (12–15)	14 (13–21) #	15 (13–19) ##	<0.05
**Tacrolimus dosing and exposure**				
Tacrolimus daily dose * [mg]	8 (5–10) ^^^	7 (5–11) ^^^	5 (3–7)	<0.001
Tacrolimus dose/kg * [mg/kg]	0.11 (0.08–0.15) ^^^	0.10 (0.07–0.16) ^^^	0.07 (0.04–0.11)	<0.01
Tacrolimus through level * [ng/mL]	9.2 (7.7–11.0)	8.4 (7.2–9.7)	9.3 (7.9–11.6)	0.37
Tacrolimus 3h post-dose level * [ng/mL]	18.2 (16.2–20.3) ^^	20.0 (16.5–22.5) ^^	14.9 (11.4–19.6)	<0.05
Tacrolimus AUC * [ng.h/mL]	149 (137–168)	156 (133–170)	137 (113–171)	0.20
Tacrolimus C/D ratio *	1.20 (0.84–1.68) ^^^	1.17 (0.77–1.70) ^^^	1.93 (1.29–3.00)	<0.001

Data presented as means with 95% confidence interval; * medians with Q1–Q3 values or frequencies, as appropriate. Statistics: ^^ *p* < 0.01, ^^^ *p* < 0.001 vs. the Envarsus group; # *p* < 0.05, ## *p* < 0.01 vs. the Prograf group. BMI, body mass index; IL-2 RB, interleukin-2 receptor blocker; ATG, antithymocyte globulin; CIT, cold ischemia time; DGF, delayed graft function; AUC, area under the curve; C/D, concentration-to-dose.

**Table 2:** The associations between recipient age and tacrolimus dosing as well as AUC according to the different tacrolimus formulations.

	Whole Study Group N = 165	Prograf Group N = 56	Advagraf Group N = 59	Envarsus Group N = 50
Tc daily dose [mg]	R = −0.332; *p* < 0.001	NS	R = −0.344; *p* < 0.01	R = −0.599; *p* < 0.001
Tc dose per body kg [mg/kg]	R = −0.392; *p* < 0.001	R = −0.297; *p* < 0.01	R = −0.397; *p* < 0.01	R = −0.603; *p* < 0.001
AUC [ng.h/mL]	R = −0.262; *p* < 0.001	R = −0.302; *p* < 0.05	R = −0.269; *p* < 0.05	NS

Correlations calculated according to Spearman. Tc, tacrolimus; AUC, area under the curve; NS, not significant.

**Table 3:** Results of bioimpedance body composition analysis and markers of intestinal permeability at hospital discharge.

	Prograf N = 56	Advagraf N = 59	Envarsus N = 50	*p*
**Body composition parameters**				
Body weight [kg]	71.1 (66.3–74.0)	73.9 (69.6–78.2)	73.7 (69.8–77.7)	0.33
Body fat mass [%]	22.9 (20.5–25.2)	25.5 (23.2–27.9)	25.5 (22.7–28.3)	0.63
ICW * [L]	23.7 (19.5–28.1)	24.5 (20.6–28.1)	24.0 (20.4–28.0)	0.81
ECW * [L]	15.5 (12.6–18.0)	16.4 (13.3–18.4)	15.6 (13.4–17.8)	0.68
ECW/TBW	0.385 (0.391–0.400)	0.384 (0.391–0.399)	0.383 (0.390–0.398)	0.80
Phase angle [^o^]	4.7 (4.5–5.0)	4.6 (4.4–4.8)	4.8 (4.5–5.1)	0.56
VFA [cm^2^] *	84.1 (48.5–104.2)	89.0 (61.8–129.7)	98.8 (56.9–125.5)	0.60
LBM [%]	72.4 (70.2–74.6)	70.1 (67.8–72.3)	70.0 (67.4–72.6)	0.24
LBMI [kg/m^2^]	18.3 (17.5–19.1)	18.4 (17.8–19.1)	18.5 (17.8–19.2)	0.95
**Markers of intestinal permeability**				
FABP-2 [ng/mL] *	1.8 ^^ (1.1–2.5)	1.4 (0.83–1.9)	1.1 (0.6–1.7)	<0.01
LPS [ng/mL] *	27.8 (23.0–33.2)	28.8 (22.5–38.0)	28.8 (22.5–39.0)	0.75
LBP [µg/mL] *	5.8 ^ (4.9–6.6)	5.1 (3.1–6.6)	4.5 (3.8–6.3)	<0.05
IL-6 [pg/mL] *	5.4 (2.9–8.6) #	3.6 (2.1–5.6)	4.1 (2.6–7.3)	0.1

Data presented as means with a 95% confidence interval; * medians with Q1–Q3 values, as appropriate. Statistics: ^ *p* < 0.05, ^^ *p* < 0.01 vs. the Envarsus group; # *p* < 0.05 vs. the Advagraf group. ICW, intracellular water; ECW, extracellular water; TBW, total body water; VFA, visceral fat area; LBM, lean body mass; LBMI, lean body mass index. FABP-2, intestinal fatty acid binding protein; LPS, lipopolysaccharide; LBP, LPS-binding protein; IL-6, interleukin-6.

**Table 4:** The correlations between body composition parameters and tacrolimus exposure measures.

	Daily Dose [mg]	Dose per kg [mg/kg]	Trough Level [ng/mL]	3h Post-Dose Level [ng/mL]	AUC [ng.h/mL]	C/D Ratio
**ECW/TBW**						
Prograf	-	-	-	R = −0.331; *p* < 0.02	R = −0.340; *p* < 0.02	-
Advagraf	-	-	-	-	-	-
Envarsus	-	-	-	-	-	-
**ECW [L]**						
Prograf	R = 0.334; *p* < 0.02	-	-	-	-	R = −0.384; *p* < 0.01
Advagraf	-	R = −0.292; *p* < 0.05	-	-	-	-
Envarsus	-	-	-	-	-	-
**ICW [L]**						
Prograf	R = 0.357; *p* < 0.01	-	-	-	-	R = −0.380; *p* < 0.01
Advagraf	-	R = −0.287; *p* < 0.05	-	-	-	-
Envarsus	-	-	-	-	-	-
**LBM [%]**						
Prograf	R = 0.267; *p* < 0.05	R = 0.275; *p* < 0.05	-	-	-	R = −0.307; *p* < 0.05
Advagraf	-	R = 0.351; *p* < 0.01	-	-	-	-
Envarsus	-	R = 0.333; *p* < 0.02	-	-	-	-
**Phase angle [°]**						
Prograf	-	-	-	R = 0.280; *p* < 0.05	R = 0.235; *p* = 0.08	-
Advagraf	-	-	-	-	-	-
Envarsus	R = 0.28; *p* < 0.05	-	-	-	-	-
**LBMI [kg/m2]**						
Prograf	R = 0.371; *p* < 0.01	-	-	-	-	R = −0.446; *p* < 0.001
Advagraf	-	R = −0.314; *p* < 0.02	-	-	-	-
Envarsus	-	-	-	-	-	-
**VFA [cm2]**						
Prograf	-	R = −0.347; *p* < 0.01	-	-	-	-
Advagraf	-	R = −0.504; *p* < 0.001	-	-	-	-
Envarsus	R = −0.293; *p* < 0.05	R = −0.453; *p* < 0.001	-	-	-	-

Correlation coefficients calculated according to Spearman. ECW, extracellular water; TBW, total body water; ICW, intracellular water; LBM, lean body mass; LBMI, lean body mass index; VFA, visceral fat area.

## Data Availability

The raw data supporting the conclusions of this article will be made available by the authors on request.
